# A randomized trial of supplemental parenteral nutrition in underweight and overweight critically ill patients: the TOP-UP pilot trial

**DOI:** 10.1186/s13054-017-1736-8

**Published:** 2017-06-09

**Authors:** Paul E. Wischmeyer, Michel Hasselmann, Christine Kummerlen, Rosemary Kozar, Demetrios James Kutsogiannis, Constantine J. Karvellas, Beth Besecker, David K. Evans, Jean-Charles Preiser, Leah Gramlich, Khursheed Jeejeebhoy, Rupinder Dhaliwal, Xuran Jiang, Andrew G. Day, Daren K. Heyland

**Affiliations:** 1 Department of Anesthesiology and Surgery and Duke Clinical Research Institute, Duke University School of Medicine, DUMC, Box 3094, Mail # 41, 2301 Erwin Road, 5692 HAFS, Durham, NC 27710 USA; 2 Faculté de Médecine de l’Université de Strasbourg, Centre Hospitalier Universitaire de Strasbourg Nouvel Hȏpital Civil, Strasbourg Cedex, France; 3 Shock Trauma Center, University of Maryland Medical Center, University of Maryland, Baltimore, MD USA; 4 Department of Critical Care Medicine, Faculty of Medicine and Dentistry, University of Alberta, Edmonton, AB Canada; 5 Divisions of Gastroenterology and Critical Care Medicine, University of Alberta Hospital, University of Alberta, Edmonton, AB Canada; 6 Division of Pulmonary, Critical Care, Allergy & Sleep Medicine, Ohio State University Medical Center, Ohio State University, Columbus, OH USA; 7 Department of Surgery, The Ohio State University Medical Center, Ohio State University, Columbus, OH USA; 8 Department of Intensive Care, Universite Libre de Bruxelles, Erasme University Hospital, Brussels, Belgium; 9 Department of Medicine, Division of Gastroenterology, University of Alberta Hospital, University of Alberta, Edmonton, AB Canada; 10 Dept. of Gastrenterology, University of Toronto, Toronto, ON Canada; 11 Clinical Evaluation Research Unit, Kingston General Hospital, Queen’s University, Kingston, ON Canada; 12 Department of Critical Care Medicine, Kingston General Hospital, Queen’s University, Kingston, ON Canada; 13 Department of Community Health and Epidemiology, Kingston General Hospital, Queen’s University, Kingston, ON Canada

**Keywords:** Parenteral Nutrition, Malnutrition, Critical care, Quality of life, Intensive care, Protein, Calorie delivery

## Abstract

**Background:**

Nutrition guidelines recommendations differ on the use of parenteral nutrition (PN), and existing clinical trial data are inconclusive. Our recent observational data show that amounts of energy/protein received early in the intensive care unit (ICU) affect patient mortality, particularly for inadequate nutrition intake in patients with body mass indices (BMIs) of <25 or >35. Thus, we hypothesized increased nutrition delivery via supplemental PN (SPN) + enteral nutrition (EN) to underweight and obese ICU patients would improve 60-day survival and quality of life (QoL) versus usual care (EN alone).

**Methods:**

In this multicenter, randomized, controlled pilot trial completed in 11 centers across four countries, adult ICU patients with acute respiratory failure expected to require mechanical ventilation for >72 hours and with a BMI of <25 or ≥35 were randomized to receive EN alone or SPN + EN to reach 100% of their prescribed nutrition goal for 7 days after randomization. The primary aim of this pilot trial was to achieve a 30% improvement in nutrition delivery.

**Results:**

In total, 125 patients were enrolled. Over the first 7 post-randomization ICU days, patients in the SPN + EN arm had a 26% increase in delivered calories and protein, whereas patients in the EN-alone arm had a 22% increase (both *p* < 0.001). Surgical ICU patients received poorer EN nutrition delivery and had a significantly greater increase in calorie and protein delivery when receiving SPN versus medical ICU patients. SPN proved feasible to deliver with our prescribed protocol. In this pilot trial, no significant outcome differences were observed between groups, including no difference in infection risk. Potential, although statistically insignificant, trends of reduced hospital mortality and improved discharge functional outcomes and QoL outcomes in the SPN + EN group versus the EN-alone group were observed.

**Conclusions:**

Provision of SPN + EN significantly increased calorie/protein delivery over the first week of ICU residence versus EN alone. This was achieved with no increased infection risk. Given feasibility and consistent encouraging trends in hospital mortality, QoL, and functional endpoints, a full-scale trial of SPN powered to assess these clinical outcome endpoints in high-nutritional-risk ICU patients is indicated—potentially focusing on the more poorly EN-fed surgical ICU setting.

**Trial registration:**

NCT01206166

**Electronic supplementary material:**

The online version of this article (doi:10.1186/s13054-017-1736-8) contains supplementary material, which is available to authorized users.

## Background

Worldwide, there is considerable controversy about the optimal amount and feeding route in critically ill patients [1]. Nutrition practice guidelines in Europe, Canada, and the United States endorse enteral nutrition (EN) for patients who are critically ill and hemodynamically stable [2–4]. To evaluate the success of EN delivery in the intensive care unit (ICU), a recent observational cohort study of nutrition practices in 167 ICUs across 21 countries was conducted to evaluate worldwide nutrition practices in 2772 patients [5]. Despite multiple international guidelines recommending early initiation of EN in the ICU [2, 3, 6], the data revealed practitioners are only successfully delivering approximately 50% of prescribed daily calories from EN over the first 12 days in the ICU [5]. In addition, in some developed countries like the United States, it takes an average of >60 hours to initiate EN [5].

Because of this consistent and longstanding failure to deliver prescribed EN, parenteral nutrition (PN) has been utilized in up to 35–70% [5] of critically ill patients. However, current guidelines do not agree on when to initiate PN in the ICU [1]. For patients who are intolerant to or have other contraindications to EN, European Society for Clinical Nutrition and Metabolism (ESPEN) guidelines recommend initiating PN within 24–48 hours in patients not expected to receive full oral nutrition within 3 days, and initiating supplemental PN (SPN) if EN levels are not at goal in 48 hours [7]. New US (American Society for Parenteral and Enteral Nutrition [ASPEN]/Society of Critical Care Medicine [SCCM]) guidelines hesitate to recommend early PN in the ICU, with PN initiation advised only after 7 days in well-nourished patients [4]. Although, in patients found to be significantly malnourished via nutrition risk scores (i.e., Nutrition Risk in Critically Ill [NUTRIC] score [without IL-6] ≥5 or Nutrition Risk Score [NRS] ≥5) [8], total PN is recommended to start at ICU admission [4, 7].

Thus, current guidelines and even recent larger randomized trials are conflicting and do not provide clear guidance regarding the use of PN in the early phase of critical illness [1]. In our previous international, multicenter, observational study, we found a significant inverse linear relationship between the odds of mortality and total daily calories received [9]. Our key finding was that increased amounts of calories were associated with reduced mortality for the body mass index (BMI) <25 group and BMI >35 group, with no benefit of increased calorie intake for patients in the BMI 25– < 35 group. Independent of the route of delivery (either EN or PN), an additional 1000 kcals was associated with an almost 50% reduction of 60-day mortality in patients with a BMI of <25 or >35 [9]. These categories of patients have not been studied separately in large-scale prospective randomized controlled trials comparing two nutritional intake levels [10–14].

Thus, we proposed a randomized trial of supplemental parenteral nutrition in underweight and overweight critically ill patients (the TOP-UP trial) as a multicenter study of critically ill underweight and obese patients with acute respiratory failure expected to require mechanical ventilation for >72 hours. In a future full trial, we proposed to address two questions: (1) the effect of SPN + EN compared with EN alone on 60-day mortality, and (2) the effect of early SPN protein and calorie intake on key quality-of-life (QoL) and functional outcomes. We estimated conservatively that a sample size of approximately 1000 patients/arm would be required to demonstrate a significant mortality effect, assuming an additional 1000 kcal/d would be associated with an approximately 29% relative risk reduction of mortality. (This was based on our pre-existing international nutrition survey data [9].) Prior to implementation of a large-scale definitive trial, we felt a multicenter pilot trial to evaluate the feasibility of a full trial was needed. The primary aim of the pilot trial reported herein was to ensure a clinically significant difference in calorie/protein intake (approximately 30% difference; or 600–1000 kcal/day and 20–30 g protein/day) between the two intervention groups was achievable. We also evaluated the feasibility of performing functional endpoints research in the ICU setting. All clinical endpoints proposed to be assessed in a future full trial were also collected and evaluated. We believe the results and experience gained from this multicenter pilot trial will allow for refinement and optimization of a full-scale multicenter trial to assess optimal methods for targeting SPN to nutritionally “at-risk” patients and inform current practice on the use of PN in the ICU.

## Methods

This was an investigator-initiated, multicenter, randomized, controlled, pilot clinical study (ClinicalTrials.gov identifier NCT01206166). This trial was conducted between June 1, 2011, and January 20, 2015, in 11 ICUs in Canada, the United States, Belgium, and France. Local jurisdictional approval and institutional research ethics board approval was secured at each site, as described in declarations section below. Written informed consent was obtained from patients, family members, or their legal representatives before enrollment. Eligible patients were randomized within 72 hours of admission to the ICU. A centralized web-based randomization system at the Clinical Evaluation Research Unit (CERU) at Kingston General Hospital was used to randomly allocate patients to study groups. Randomization was stratified by site, presence of medical or surgical admission diagnosis, EN started before randomization, and BMI (<25 or >35). Patients were randomized in random block sizes of two, four, or eight within strata.

### Trial participants

Consecutive mechanically ventilated adults admitted to participating ICUs were screened for eligibility. Critically ill adult patients (>18 years old) in the ICU were considered eligible for the study if they met the following criteria: (1) had acute respiratory failure (defined as expected to require mechanical ventilation >72 hours), (2) were receiving EN or were to be initiated on EN within 48 hours of ICU admission, and (3) had a BMI of <25 or >35, based on pre-ICU actual or estimated dry weight. Exclusions included the following: (1) >72 hours from ICU admission to consent, (2) not expected to survive an additional 48 hours from screening evaluation, (3) lack of commitment to full, aggressive care (anticipated withholding or withdrawing treatments in the first week, but isolated do-not-resuscitate order acceptable), (4) an absolute contraindication to EN deemed to require PN for the first 7 days of ICU admission (e.g., gastrointestinal obstruction or no gastrointestinal tract access for any reason), (5) already at goal rate of EN from screening evaluation (receiving ≥60% estimated needs and no evidence of intolerance [i.e., high gastric residual volumes, etc.]), (6) already receiving PN on admission to ICU, (7) admitted with diabetic ketoacidosis or nonketotic hyperosmolar coma, (8) pregnant or lactating, (9) clinical fulminant hepatic failure, (10) dedicated port of central line not available, (11) known allergy to study nutrients, and (12) enrollment in another industry-sponsored ICU intervention study (co-enrollment in academic studies were considered on a case-by-case basis).

### Trial interventions

Patients were randomized to receive either EN (standard care) or SPN + EN. The type of enteral formula was selected by the individual treatment team following nutritional assessment. A standard polymeric solution with 1.2 ± 0.2 kcal/mL was used to standardize nutrition delivery. EN was initiated at 20 mL/hr and increased by 20 mL/hr increments every 4 hours as tolerated until the goal rate was reached. A bedside algorithm was developed to aid in initiating and progressing the EN rate.

In patients assigned to the SPN + EN group, all patients received SPN via central intravenous access and SPN administration began as soon as possible post-enrollment. We utilized a PN solution of similar caloric density to the standard EN solutions (1.2 kcals/mL, providing 0.06–0.09 g protein/mL). The PN solution utilized (OLIMEL N9, Baxter Inc., Deerfield, IL, USA) was a 1.1 kcal/mL solution—20% lipid (containing 80% olive oil and 20% soy oil), 27.5% glucose solution, and 14% amino acids. PN was initiated at 20 mL/hr and increased by 20 mL/hr increments every 4 hours as tolerated until 100% of goal calories were reached. The PN was adjusted daily to ensure that patients received 100% of their prescribed calories.

In both groups, the relative amount of PN and EN received was monitored. All patients were fed according to the Canadian Critical Care Nutrition 2003 clinical practice guidelines [2], which are updated online (www.criticalcarenutrition.com). Blood glucose, insulin dose, dextrose infusion rates, and electrolytes were monitored frequently, as clinically indicated (at minimum daily as per the study protocol), and neither EN nor PN was advanced if electrolytes, glucose, or phosphate was critically out of range to minimize and evaluate for refeeding-syndrome risk. EN or SPN + EN were continued for 7 days post-randomization or until death, whichever came first. In extubated patients, PN and/or EN was continued until >50% of caloric goals were tolerated by oral route. In the event that a patient was discharged from the ICU prior to day 7, PN could be continued in-hospital until the patient was tolerating adequate EN or oral nutrition. At the end of the study period, clinicians could prescribe PN using the study solution (OLIMEL N9) as clinically indicated in either group.

### Nutrition prescription

Both the EN-only (control) and SPN + EN (study) groups received the exact same prescription for calories and protein (within each BMI stratum), with the study group receiving additional calories and protein via parenteral route. Upon enrollment, study dieticians calculated the protein and calorie needs of each patient. The proposed target dose of protein and energy based on BMI category is described in Table 1.

**Table 1:** Protein and energy provision: guidelines for dosing of protein and energy based on BMI category

	Minimum energy	Minimum protein
BMI <25	25 kcals/kg actual wt	1.2 g/kg actual wt
BMI >35	20 kcals/kg ABW	1.2/kg ABW

Weights in obese patients calculated according to the following formula: *obesity – adjusted body weight = IBW + [actual weight – IBW] x 0.25*, where IBW is based on a BMI of 25

*Abbreviation*s: *ABW*, adjusted body weights, *BMI* body mass index, *IBW*, ideal body weight

### Outcomes

The primary dual outcome for this pilot trial was to achieve an increased calorie and protein delivery (by approximately 30%) in the SPN + EN group versus EN alone. We also analyzed calorie and protein delivery in patients with BMIs <25/>35 and in surgical ICU patients versus medical ICU patients, as our previous data indicated surgical ICU patients were more poorly fed than other ICU groups [15]. Secondary outcomes included testing the feasibility of implementing the SPN intervention, quality measures regarding protocol adherence, and success in intervention delivery. Additional outcomes included ICU, hospital, and 6-month mortality; development of infectious complications; and duration of mechanical ventilation, ICU stay, and hospital stay. In addition, functional indices were assessed, including admission and discharge Barthel Index, handgrip strength, and 6-minute walk test at discharge. At 3 and 6 months post-randomization, patients were contacted by telephone to record vital status and SF-36 (36-Item Short Form Health Survey) scores.

### Subgroup analyses

We explored several pre-specified subgroups. Sicker patients with objectively defined high nutrition risk may benefit more from nutritional interventions (as defined by a NUTRIC score without IL-6 ≥ 5) [8]. Thus, patients with increased NUTRIC scores (≥5) versus lower scores were compared for ICU and hospital mortality. Further, as one admission BMI group (<25 or ≥35) may benefit more from nutrition interventions than the other, these two groups were also compared for ICU and hospital mortality.

### Statistical analysis

The sample size for this pilot trial was targeted to assess the feasibility of an international study and provide adequate precision to estimate the difference of nutritional adequacy between groups. In particular, given the observed evaluable sample size (71 EN only and 49 SPN + EN) and standard deviation, the difference of all nutritional adequacy measures were estimated to within 10% with at least 95% certainty. The dual primary endpoint was the proportion of caloric and protein prescription received by EN or PN, including protein supplements but excluding propofol. This proportion is based only on days after the date of randomization and before the date of death or ICU discharge where oral feeding did not preclude the use of EN or PN. The proportions of caloric and protein prescriptions delivered were presented within groups by means and standard deviations and compared between groups by mean differences with 95% confidence intervals (CIs) and *p* values estimated by the two-sample *t* test for unequal variances. Averages over the first 7 days after randomization (primary) and 27 days after randomization (secondary) were presented.

ICU and hospital mortality are described within groups as counts and percentages and were compared between groups by the chi-squared test. Furthermore, hospital mortality was compared between groups overall and within subgroup by odds ratios (ORs) with 95% CIs. Median 6-month survival was estimated within group by the Kaplan-Meier method and compared between groups by the log-rank test.

All other continuous or ordinal variables were compared between groups by the rank-based Mann-Whitney *U* test. The handgrip and 6-minute walk tests were ranked as follows: died < unable < refused = 0 < other non-zero values, with patients whose assessment was missed being excluded. Barthel Index and SF-36 scores were based only on patients with values available, and thus excluded decedents and those lost to follow-up. Infection outcomes were presented by groups as counts and percentages, with patient-level summaries compared between groups by Fisher’s exact test.

With the exceptions of the aforementioned exclusions, patients were analyzed as randomized regardless of treatment compliance in accordance with the intent-to-treat principle. We did not attempt to impute unknown values or correct for multiplicity due to the primarily exploratory descriptive nature of this pilot feasibility study.

## Results

**Fig. 1:**
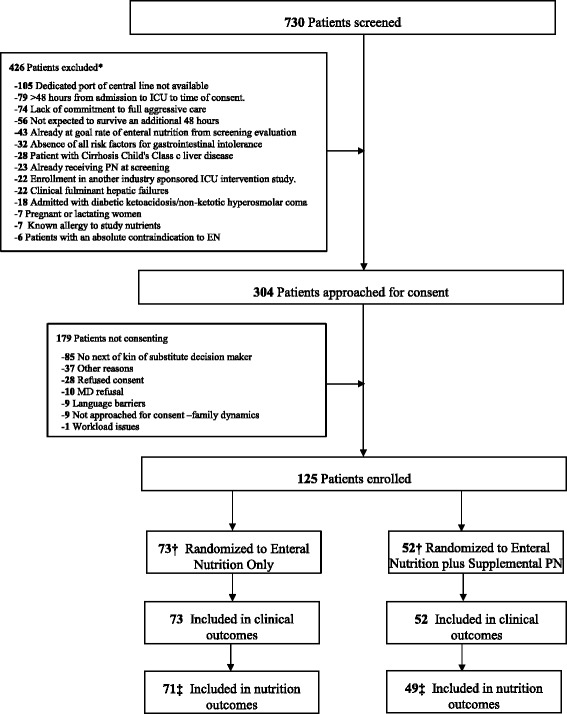
Patient flow diagram. * Exclusion reasons add up to greater than 426 because some patients have multiple exclusion reasons. †The large imbalance between arms is purely due to chance. This imbalance was possible despite the blocked randomization due to the large number of strata with incomplete blocks. ‡ Two EN and three EN+PN patients had no days evaluable for nutritional adequacy due to not having any days after randomization and before discharge or death without oral feeding

Over a 44-month recruitment period, 730 patients were screened, of whom 304 met enrollment criteria and 125 were randomized (Fig. 1). Screening periods at sites varied; enrollment was capped after 20 patients to allow for other sites to contribute. Overall, the average enrollment rate per site was 0.8 patients/month (range 0.3–1.9). Characteristics at baseline were similar in both groups (Table 2). Quality measures regarding protocol adherence and success in intervention delivery are reported in Table 3. Overall, patients in the SPN + EN group were randomized and initiated on study PN rapidly after ICU admission and had a median study intervention duration of 5.9 days (range 2.4–7.6). In the SPN + EN group, 13 patients (25%) received <80% of goal calories/day at some point during their enrollment in trial, which was reported as a protocol violation (Table 4). The reasons for these episodes of <80% of goal calories being delivered during a given day are reported in Table 5. In total, 16 patients (30.8%) in the SPN + EN arm received <72 hours of study PN, and 3 of these patients never received SPN because their nutritional goal was reached early by EN alone.

**Table 2:** Patient demographics

Characteristic	EN only (*n* = 73)	SPN + EN (OLIMEL) (*n* = 52)
Age, yrs	55.1 ± 16.2	55.8 ± 19.8
Sex		
Male	39 (53.4%)	21 (40.4%)
Female	34 (46.6%)	31 (59.6%)
APACHE II score	20.8 ± 7.2	20.5 ± 6.4
Baseline SOFA score	5.9 ± 3.6	6.2 ± 3.5
NUTRIC score	3.8 ± 2.1	3.9 ± 1.9
Barthel Index baseline	88.1 ± 22.7	91.3 ± 11.7
BMI	33.2 ± 15.0	33.5 ± 14.9
BMI groups		
< 25	38 (52.1%)	27 (51.9%)
> 35	35 (47.9%)	25 (48.1%)
Ethnicity		
White	65 (89.0%)	46 (88.5%)
Black or African American	4 (5.5%)	2 (3.8%)
Native Hawaiian or Pacific Islander	2 (2.7%)	0 (0.0%)
Native	2 (2.7%)	0 (0.0%)
Unknown/not reported	0 (0.0%)	4 (7.7%)
Charlson Comorbidity Index	1.7 ± 1.9	1.3 ± 1.7
Type of admission		
Medical	43 (58.9%)	31 (59.6%)
Surgical	30 (41.1%)	21 (40.4%)
Primary diagnosis		
Respiratory	24 (32.9%)	13 (25.0%)
Sepsis	18 (24.7%)	15 (28.8%)
Gastrointestinal	9 (12.3%)	6 (11.5%)
Neurologic	9 (12.3%)	5 (9.6%)
Other	4 (5.5%)	1 (1.9%)
Trauma	3 (4.1%)	0 (0.0%)
Metabolic	3 (4.1%)	0 (0.0%)
Cardiovascular/vascular	3 (4.1%)	11 (21.2%)
Hematologic	0 (0.0%)	1 (1.9%)

Continuous variables are reported as mean ± standard deviation, and categorical variables are reported as count (% of column total)

*Abbreviations*: *APACHE II* Acute Physiology and Chronic Health Evaluation II, *BMI* body mass index, *EN* enteral nutrition, *NUTRIC* Nutrition Risk in Critically Ill, *SOFA* Sequential Organ Failure Assessment, *SPN* supplemental parenteral nutrition

**Table 3:** Primary outcome: calorie and protein delivery

	EN only (*n* = 71)	SPN + EN (OLIMEL) (*n* = 49)	Difference mean, % (95% CI)	*p* value
Evaluable days	11 ± 7	11 ± 8	0 (−2 to 3)	0.765
Evaluable days in first week	6 ± 2	6 ± 2	0 (−1 to 1)	0.992
Calorie prescription	1844 ± 420	1728 ± 444	−116 (−275 to 42)	0.149
Protein prescription	106 ± 30	100 ± 31	−6 (−17 to 6)	0.319
% of prescribed kcal/protein received				
EN only				
Calories first 27 days	70 ± 26	67 ± 25	−3 (−12 to 7)	0.551
Calories first 7 days	68 ± 28	68 ± 27	−1 (−11 to 9)	0.905
Protein first 27 days	66 ± 26	60 ± 23	−5 (−14 to 3)	0.231
Protein in first 7 days	63 ± 26	61 ± 25	−3 (-12 to 7)	0.566
PN + EN				
Calories first 27 days	72 ± 25	90 ± 16	18 (11 to 25)	<0.001
Calories first 7 days	69 ± 28	95 ± 13	26 (18 to 34)	<0.001
Protein first 27 days	68 ± 25	82 ± 19	13 (6 to 21)	<0.001
Protein in first 7 days	64 ± 26	86 ± 16	22 (14 to 29)	<0.001

Values are means ± standard deviations, unless noted otherwise. *P* values and 95% CIs were calculated by the independent *t* test for unequal variance. Only days after the date of randomization and before date of ICU discharge or death are considered evaluable days. Days where oral feeding was indicated as the reason for not receiving EN or PN have also been excluded. Two patients randomized to the EN arm and three patients randomized to the SPN + EN arm had no evaluable days and are thus excluded from this analysis. All calories exclude propofol but include protein supplementation. PN includes both study PN and non-study PN

*Abbreviations*: *CI* confidence interval, *EN* enteral nutrition, *PN* parenteral nutrition, *SPN* supplemental parenteral nutrition

**Table 4:** Primary outcome quality measures: intervention

Variable	EN only (*n* = 73)	SPN + EN (OLIMEL) (*n* = 52)	*p* value
Days from ICU admission to randomization	1.4 (0.8–2.0)	1.1 (0.7–1.8)	0.19
Hours from randomization to start of intervention (SPN + EN arm)	—	1.6 (0.6–4.9)	—
Duration of intervention, days (SPN + EN arm)	—	5.9 (2.4–7.6)	—
Protocol violation: <80% study PN (SPN + EN arm)	—	13 (25.0%)	—
Protocol violation: >120% study PN (SPN + EN arm)	—	2 (3.8%)	—
Other protocol violations and reasons			
Received non-study PN before 7 days	5 (6.8%)	0 (0.0%)	0.05
Received non-study IV lipids before 7 days	1 (1.4%)	0 (0.0%)	0.40
Received protein supplements before 7 days	1 (1.4%)	4 (7.7%)	0.08
Received study PN before 7 days (EN-only arm)	2 (2.7%)	—	—
Other (no further data provided)	1 (1.4%)	0 (0.0%)	0.40
Early deaths or drop-outs^a^ (<72 hrs on protocol)	1 (1.4%)	10 (19.2%)	0.16

Data reported as median (Q1–Q3) or n (%). The Mann-Whitney *U* test was used for continuous variables, and the chi-square test was used for categorical variables

*Abbreviations*: *EN* enteral nutrition, *ICU* intensive care unit, *IV* intravenous, *PN* parenteral nutrition, *SPN* supplemental parenteral nutrition

^a^This occurred in the PN group due to the following reasons: goal was reached by EN-alone group in 72 hours (*n* = 6), transitioned to oral feeds (*n* = 2), central line removed (*n* = 1), and fluid overload (*n* = 1)

**Table 5:** Reasons for protocol violation of patients receiving <80% volume in SPN + EN group)

Reason(s)	Counts
Nausea/emesis/patient too sick	30
Unknown/error	17
First or last day of EN, including withdrawal of care	16
No access/held for procedure	12
High gastric residuals	6
On oral feeds	6
Total episodes leading to <80% of volume in SPN + EN group	87

*Abbreviation*: *EN* enteral nutrition, *SPN* supplemental parenteral nutrition

### Primary outcome: delivery of calories and protein

**Fig. 2:**
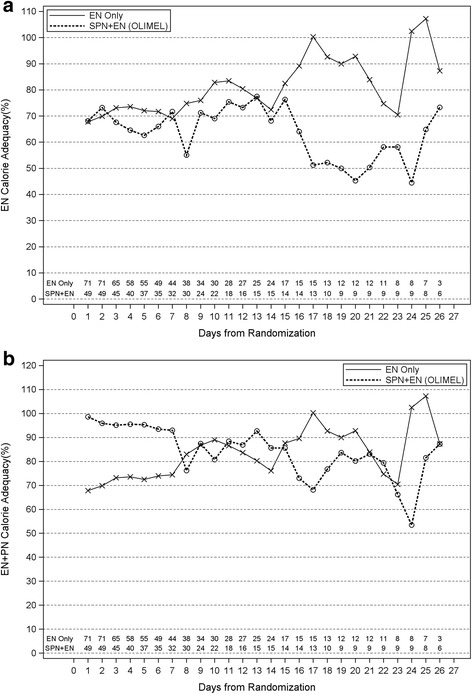
ICU calorie adequacy. **a** EN calorie adequacy. **b** EN + PN calorie adequacy. ○ - SPN + EN group, X - EN alone group. The number of patients in each group on each day of the study is shown at the bottom of the graphs. *EN* enteral nutrition, *PN* parenteral nutrition, *SPN* supplemental parenteral nutrition

Three patients in the SPN + EN group and one patient in the EN-alone group were excluded from the analysis of nutrition delivery because they had no days after the date of randomization and before the date of ICU discharge or death where EN and PN were not precluded due to oral feeding (Fig. 1). Over the first 7 days after randomization, patients in the SPN + EN arm had increases in calorie and protein delivery of 26% and 22%, respectively, versus EN alone (both *p* < 0.001) (Table 3 and Fig. 2). Over the first 27 days after randomization, patients in the SPN + EN arm had increases in calories and protein delivery of 18% and 13%, respectively (both *p* < 0.001; Table 3 and Fig. 2). Surgical ICU patients had a significant increase in calorie and protein delivery versus medical ICU patients in the SPN + EN arm (38% vs. 18% and 35% vs. 13%, respectively) (*p* < 0.05) (Additional file 1: Table S1A and S1B). High BMI (>35) patients had a small increase of calorie and protein delivery versus low BMI (<25) patients (31% vs. 21% and 25 vs. 18%, respectively); however, these differences were not statistically significant. (Additional file 1: Table S2A and S2B).

**Fig. 3:**
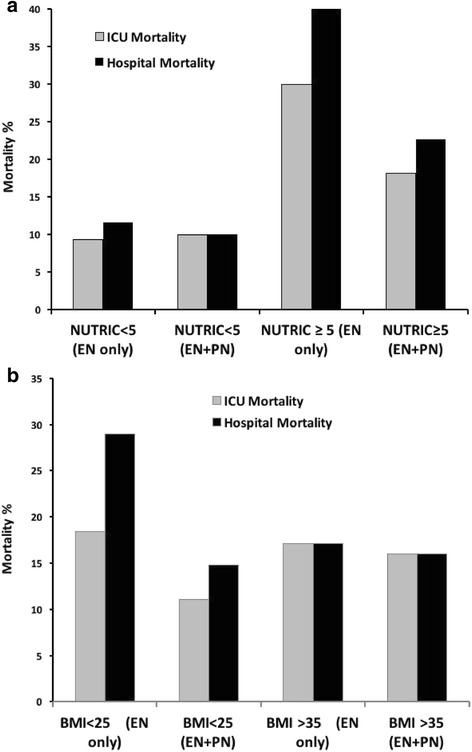
Hospital and ICU mortality outcomes by subgroup. **a** Mortality outcomes by admit NUTRIC score <5 (n = 73) and >5 (n = 52). **b** Mortality outcomes by BMI <25 (*n* = 65) and >35 (*n* = 60). Odds ratio for hospital mortality by subgroup. *BMI* body mass index, *EN* enteral nutrition, *ICU* intensive care unit, *PN* parenteral nutrition

### Clinical outcomes

Although this pilot trial was not powered primarily for clinical outcomes, assessment of clinical outcome differences between groups was undertaken to help guide definitive trial design and assess for clinical signals justifying a larger definitive trial. No significant difference in major clinical outcomes between groups was observed (Table 6). This included no increased rate of suspected or newly acquired infections in the SPN + EN group versus the EN-alone group (Table 7). A somewhat lower hospital mortality was observed in the SPN + EN group versus the EN-alone group, although this was not statistically significant (OR 0.60, 95% CI 0.24–1.52; *p* = 0.28). Potential trends were observed for reduced hospital mortality in the SPN + EN group versus the EN-alone group in high-nutritional-risk patients (both NUTRIC ≥5 and in BMI <25; *p* = 0.19; Fig. 3. No apparent differences in mortality were observed in patients with a BMI ≥35 or NUTRIC score <5 (Fig. 3).

**Table 6:** Clinical outcomes

Variable	EN only (*n* = 73)	SPN + EN (OLIMEL) (*n* = 52)	*p* value
Length of ventilation, days	8.3 (3.8–13.3)	6.5 (3.9–14.1)	0.78
ICU mortality			0.51
Yes	13 (17.8%)	7 (13.5%)	
No, patient discharged	60 (82.2%)	45 (86.5%)	
Length of stay in ICU among survivors, days	12.6 (8.1–18.7)	12.8 (7.9–17.8)	0.80
Hospital mortality			0.29
Yes	17 (23.3%)	8 (15.4%)	
No, patient discharged	56 (76.7%)	43 (82.7%)	
No, patient still in hospital at 6 months	0 (0.0%)	1 (1.9%)	
Length of stay in hospital among survivors, days	24.0 (16.6–38.9)	23.5 (17.5–34.7)	0.83
Time to discharge alive from hospital	33.0 (20.2, und)	32.5 (21.1, und)	0.87
Kaplan-Meier 6-month mortality estimate^a^	27.5%	29.5%	0.86

Data reported as median (Q1–Q3) or n (%). The Mann-Whitney *U* test was used for continuous variables, and the chi-square test was used for categorical variables. Survival analysis was used for comparison of time to discharge alive from hospital

*Abbreviations*: *EN* enteral nutrition, *ICU* intensive care unit, *SPN* supplemental parenteral nutrition, *und* undefined due to <75% reaching upper quartile

^a^The Kaplan-Meier estimate censors patients at the last known date alive. Altogether, 19 deaths were observed in the EN-alone arm, compared with 14 deaths in the SPN + EN arm. The median follow-up time among patients where death was not observed was 175 days in the EN-alone arm and 167 days in the SPN + PN arm

**Table 7:** Infection outcomes

Variable	EN only (*n* = 73)	SPN + EN (OLIMEL) (*n* = 52)	*p* value
Number of patients with a suspected infection	33/73 (45.2%)	26/52 (50.0%)	0.72
Total number of suspected infections	83	78	
Average suspected infections per patient, ± SD	1.7 ± 2.6	1.9 ± 2.6	0.62
Number of patients with newly acquired infection	23/73 (31.5%)	14/52 (26.9%)	0.69
Total number of newly acquired infections	46	38	
Adjudication^a^			
Definite	30 (65.2%)	18 (47.4%)	
Possible	1 (2.2%)	2 (5.3%)	
Probable	15 (32.6%)	18 (47.4%)	
Type of newly acquired infection^a^			
Surgical deep	0 (0.0%)	1 (2.6%)	
Skin/soft tissue	3 (6.5%)	0 (0.0%)	
Catheter BSI	0 (0.0%)	7 (18.4%)	
Primary BSI	1 (2.2%)	0 (0.0%)	
Lower UTI	2 (4.3%)	5 (13.2%)	
Upper UTI	0 (0.0%)	1 (2.6%)	
Intra-abdominal	0 (0.0%)	4 (10.5%)	
Lower RTI	17 (37.0%)	5 (13.2%)	
ICU pneumonia	18 (39.1%)	12 (31.6%)	
Other	5 (10.9%)	3 (7.9%)	
Organism types^b^	27	19	
Bacteria	24 (88.9%)	14 (73.7%)	
Fungi/yeast	2 (7.4%)	5 (26.3%)	
Virus	1 (3.7%)	0 (0.0%)	

Mean ± SD reported for continuous variables. Count (%) reported for categorical variables. Number of suspected and newly acquired infections was compared using the Fisher’s exact test, and the average number of infections per patient was compared using the Mann-Whitney *U* test

*Abbreviations*: *BSI* bloodstream infection, *EN* enteral nutrition, *ICU* intensive care unit, *RTI* respiratory tract infection, *SD* standard deviation, *SPN* supplemental parenteral nutrition, *UTI* urinary tract infection

^a^The denominator is the total number of newly acquired infections

^b^The denominator is the total number of newly acquired infections with organisms detected

### Functional and quality-of-life outcomes

Overall trends to improved hospital discharge handgrip strength (*p* = 0.14) and 6-minute walk test score (*p* = 0.2) were observed in SPN + EN group versus the EN-alone group (Table 8). A potential non-significant tendency to improved handgrip strength at ICU discharge in the SPN + EN group was also observed (*p* = 0.21). Trends to improved hospital discharge Barthel Index (*p* = 0.08) was also observed. Although inconsistent at 3 months, by 6 months the change in SF-36 was consistently (but not significantly) better in the SPN + EN group versus the EN-alone group. Challenges in collection of functional endpoints (Table 9) were observed; in most cases this was due to the patient being too debilitated or ill to perform the test. For example, in collecting data for the 6-minute walk test, a significant number of patients could not perform the test due to death (20%) or, more commonly, due to severity of impairment from their critical illness (40%).

**Table 8:** Functional and quality-of-life outcomes

Variable	EN only (*n* = 73)	SPN + EN (OLIMEL) (*n* = 52)	*p*-value
Handgrip at ICU discharge	Unable (62) [unable–18]	9 (43) [unable–25]	0.21
Handgrip at hospital discharge	Unable (56) [unable–20]	12 (36) [unable–33]	0.14
6-minute walk test at hospital discharge	Unable (60) [unable–unable]	Unable (40) [unable–0]	0.20
Barthel Index hospital discharge	46.5 ± 32.1 (41)	61.1 ± 32.4 (28)	0.08
SF-36 3 months			
Physical functioning	39.4 ± 34.3 (30, 55%)	34.8 ± 31.5 (24, 63%)	0.76
Role-physical	30.2 ± 31.8 (30, 55%)	32.8 ± 32.6 (25, 66%)	0.59
Pain index	59.1 ± 28.8 (28, 52%)	66.4 ± 27.3 (24, 63%)	0.44
General health perceptions	61.2 ± 18.3 (27, 50%)	49.5 ± 24.3 (24, 63%)	0.14
Vitality	52.8 ± 21.4 (28, 52%)	51.0 ± 21.7 (24, 63%)	0.72
Social functioning	60.4 ± 31.8 (30, 55%)	56.5 ± 28.2 (25, 66%)	0.56
Role-emotional	63.2 ± 34.6 (29, 54%)	65.3 ± 34.4 (25, 63%)	0.88
Mental health index	72.9 ± 18.7 (28, 52%)	76.1 ± 18.5 (23, 61%)	0.39
Standardized physical component scale	35.3 ± 10.8 (27, 50%)	33.3 ± 10.1 (22, 58%)	0.38
Standardized mental component scale	50.0 ± 10.5 (27, 50%)	51.5 ± 10.0 (22, 58%)	0.38
SF-36 6 months			
Physical functioning	39.3 ± 34.0 (31, 57%)	50.8 ± 36.5 (20, 53%)	0.21
Role-physical	40.2 ± 33.1 (32, 59%)	47.5 ± 33.4 (20, 53%)	0.43
Pain index	52.5 ± 31.0 (31, 57%)	68.6 ± 28.2 (20, 53%)	0.08
General health perceptions	50.9 ± 20.6 (31, 57%)	56.8 ± 26.2 (20, 53%)	0.46
Vitality	47.8 ± 21.2 (31, 57%)	59.1 ± 21.7 (20, 53%)	0.06
Social functioning	50.4 ± 32.2 (31, 57%)	68.8 ± 32.6 (20, 53%)	0.06
Role-emotional	52.2 ± 41.0 (32, 59%)	72.1 ± 30.3 (20, 53%)	0.10
Mental health index	66.1 ± 22.5 (31, 57%)	70.5 ± 24.9 (20, 53%)	0.36
Standardized physical component scale	35.8 ± 11.2 (30, 55%)	39.3 ± 10.2 (20, 53%)	0.17
Standardized mental component scale	43.2 ± 14.8 (30, 55%)	49.0 ± 13.5 (20, 53%)	0.11

Handgrip strength and 6-minute walk test data using rank-based analysis: Values reported as n (%) or n (median) [Q1–Q3]. (n = observations collected). Values that were missed or have an unknown reason for not being done are excluded. The remaining values are ranked as died < unable < refused = 0 < other non-zero values. The *p* values are calculated by the rank-based Wilcoxon rank-sum tests. Barthel Index and SF-36 data: ranges for Barthel Index and SF-36 are 0–100, with 100 as the best score. Mean ± SD (n = observations collected, % of possible measures that could be obtained after subtracting out deaths prior to measurement time point) was reported for continuous variables. The Mann-Whitney *U* test was used for comparisons

*Abbreviations*: *EN* enteral nutrition, *ICU* intensive care unit, *SF-36* 36-Item Short Form Health Survey, *SPN* supplemental parenteral nutrition

**Table 9:** Functional outcomes compliance: handgrip strength and 6-minute walk test

Variable	EN only (*n* = 73)	SPN + EN (OLIMEL) (*n* = 52)	*p* value
Handgrip at ICU discharge			
Patient died	13 (17.8%)	7 (13.5%)	
Unable to do	18 (24.7%)	9 (17.3%)	
Refused to do	2 (2.7%)	3 (5.8%)	
Done	29 (39.7%)	24 (46.2%)	
Missed	9 (12.3%)	6 (11.5%)	
Unknown reason	2 (2.7%)	3 (5.8%)	
Handgrip at hospital discharge			
Patient died	17 (23.3%)	8 (15.4%)	
Unable to do	12 (16.4%)	5 (9.6%)	
Refused to do	3 (4.1%)	3 (5.8%)	
Done	22 (30.1%)	17 (32.7%)	
Missed	16 (21.9%)	12 (23.1%)	
Unknown reason	1 (1.4%)	4 (7.7%)	
Use ICU	2 (2.7%)	3 (5.8%)	
6-minute walk test at hospital discharge			
Patient died	17 (23.3%)	8 (15.4%)	
Unable to do	31 (42.5%)	20 (38.5%)	
Refused to do	3 (4.1%)	3 (5.8%)	
Done	9 (12.3%)	9 (17.3%)	
Missed	12 (16.4%)	9 (17.3%)	
Unknown reason	1 (1.4%)	3 (5.8%)	
Walked on or any day prior to ICU discharge	16 (21.9%)	11 (21.2%)	0.92

Values reported as n (%)

*Abbreviations*: *EN* enteral nutrition, *ICU* intensive care unit, *SPN* supplemental parenteral nutrition

## Discussion

In this pilot trial of SPN + EN versus EN alone, we found SPN + EN significantly increased calorie/protein delivery over the first ICU week, nearly achieving the targeted 30% increase in caloric delivery. SPN + EN proved feasible to deliver with our prescribed protocol. As expected in this pilot trial, which was not powered for clinical outcomes, no significant outcome differences, including no difference in infection risk between groups, were observed. However consistent encouraging trends in hospital/ICU mortality, QoL, and functional endpoints in the SPN + EN group were observed. Signals of reduced mortality in the NUTRIC ≥5 and BMI <25 subgroups also indicate that SPN + EN may have a particular benefit in higher-nutritional-risk, lower-BMI patients.

Enrollment of critically ill patients meeting the BMI <25 or >35 criterion proved challenging. As the average BMI in recent North American and even European ICU nutrition trials has ranged from 26.5–30.1 [11, 16, 17], a limited number of patients were ultimately eligible for screening. As a result of funding constraints and eligibility challenges, enrollment was constrained to 125 total patients. Further, we block-randomized patients, stratifying by site, medical/surgical diagnosis, BMI, and baseline use of EN. Since the study had several small sites and a large number of strata (eight within each site), there was a high proportion of incomplete blocks, which undermined the effectiveness of the stratification and allowed for a large overall imbalance in the number of patients randomized to each arm. This increases the variance of the between-arm comparisons by 3%, compared with if we had the same number in both arms (see Hsieh et al. for the VIF formula of (k + 1)^2/(4 k) where k = 73/52) [18]. Or, stated another way, this imbalance results in a study with the same power and precision as a study with a total sample size that is 3% smaller but has even numbers in each arm. Thus, this imbalance may have caused a minimal reduction in power but does not meaningfully or statistically bias the estimates or interfere with results. Although this would be less of an issue for a much larger trial, it may be worth considering reducing the number of strata or using an alternative balancing method such as minimization [19]. Another limitation of this study is that all calorie prescriptions were determined using weight-based formulas. Compared with indirect calorimetry-determined nutrition targets, these prescriptions may lead to a greater risk of over- or under-feeding actual caloric need [20]. In the future, we hope for improved metabolic cart availability to allow for improved guidance of feeding targets in the ICU.

Compliance with pre-discharge and post-discharge functional and QoL measures proved challenging to collect in all patients. For the functional tests, this was most often due to patients’ inability to complete testing due to death or significant disability following ICU stay. For example, 60% of patients could not complete the hospital discharge 6-minute walk test due to either an inability to walk (40%) or death (20%). The rank-based analytic approach allowed the inclusion of decedents and patients too ill to perform functional testing. This challenge in obtaining functional outcomes post-ICU stay has been observed in similar trials, such as the EPaNIC trial, where only approximately 26% of enrolled patients were able to complete or provide data at the ICU discharge 6-minute walk test [10]. The ability for patients to complete functional endpoints and rigorous follow-up for QoL outcomes requires careful consideration when designing future trials. Collection of functional outcomes continues to be a challenge for ICU trials with many patients who are too debilitated to perform many of the functional outcome measures.

Another key issue in ICU pilot trials regarding compliance with new, more complex study procedures (such as handgrip strength and 6-minute walk testing) is that other critical care trials have demonstrated that enrollment of the first one to three patients in each site is effectively a “run-in period” that can be fraught with complexity [21, 22]. These data would indicate that after the second patient is randomized, site protocol violations decrease and treatment effect tends to increase (i.e., becomes more stable toward the true estimate of treatment effect). Thus, in a larger definitive trial, compliance may improve with larger patient numbers enrolled at each site, producing more complex functional and lean body mass outcomes.

Strengths of this study include that we were able to demonstrate a significant separation in the amount of delivered calories and protein between groups with early SPN, particularly in surgical ICU patients. Other key findings include that early SPN did not contribute to any increased risk of infection, as has been hypothesized by past trials [23]. Another strength is the utilization of a more modern, non-pure-soy-oil-based lipid formulation, which may have contributed to the lack of infection risk from SPN in this trial. Recent meta-analyses have shown that lipid formulations reducing soy-based lipid delivery via use of non-pure-soy-oil formulations have lower rates of infection in ICU patients [24].

A key goal of this trial was to attempt to identify a “high nutritional risk” group of ICU patients to target the use of more complex PN delivery and assess the potential benefits of SPN + EN given poor EN delivery worldwide. In this pilot study, encouraging trends toward reduced ICU and hospital mortality were observed only in the BMI <25 subgroup of the SPN + EN arm, and no trend was observed in the BMI >35 subgroup. Thus, it is possible that this strategy of early SPN delivery may have greatest efficacy in patients with lower BMIs and who may have the lowest lean body mass reserve. As neither BMI group was powered to meaningfully look at clinical outcomes, both BMI subgroups should be considered targets of future research and will require further study. In addition, subgroup analysis revealed that patients with the highest ICU admission nutrition risk, as defined by a NUTRIC score of ≥5, appeared to show the largest trend to benefit from SPN. As such, we believe that the future full TOP-UP trial should focus enrollment on patients with a NUTRIC score ≥5 to target, or personalize, early SPN therapy for patients most likely to benefit. Thus, we may have further learned that BMI is not the ideal indicator of nutrition risk in the ICU, but perhaps the NUTRIC score has promise as a better objective measure of nutritional risk [8, 25].

Additionally, a significantly greater increase in calorie delivery was achieved by SPN + EN over EN alone in the surgical ICU patients versus medical ICU patients. As has been previously described [15], surgical ICU patients in our study had a much poorer delivery of baseline EN than the medical ICU patients. Further, the targeted greater than 30% increase in calorie delivery by SPN was also able to be achieved in the surgical ICU group. It is possible these data suggest that a future SPN trial may also be optimally focused on a high-nutritional-risk surgical ICU group, as these patients demonstrate a greater deficit in EN calorie and protein delivery and thus may be more likely to benefit from additional SPN delivery.

Finally, over the last 10 years we have begun to reduce in-hospital mortality following severe sepsis in some countries worldwide [26]. However, the same data also reveal that we have tripled the number of patients going to rehabilitation settings [26]. We also know that up to 40% of mortality within the first year of ICU stay occurs after ICU discharge [27], often due to post-intensive care syndrome (PICS). As a result, many leading experts are calling for future ICU trials to not focus on mortality as the primary endpoint, but rather to focus on QoL [26]. As such, we strived to introduce functional and key QoL indicators in our outcomes, particularly as early protein/calorie delivery may be key in optimizing post-ICU lean body mass and QoL. Our pilot data reveal consistent trends in improvement of functional and QoL endpoints in the SPN + EN group versus EN alone. In particular, trends to improved hospital discharge handgrip strength, 6-minute walk test, Barthel Index, and SF-36 scores were observed in the SPN + EN group versus EN alone. This included a significant improvement in the vitality subscore at 6 months (*p* = 0.05). Overall, these data are consistent in the direction of benefit for functional and QoL outcomes in patients receiving early SPN, and we believe this deserves further study in the larger TOP-UP trial. Further, given the consistent signal seen in functional and QoL outcomes, we would propose considering a QoL or functional outcome be the primary outcome of a future full-scale SPN trial. For, as many have said, the epidemic of PICS is one that we must address with targeted trials as soon as possible [26, 28].

## Conclusions

This pilot trial was undertaken to answer key questions on the feasibility of conducting a multinational, multicenter trial of SPN in low- and high-BMI patients, based on the concept that these patients would most likely benefit from additional calorie and protein delivery in the first week of ICU care. Additionally, compliance and patient ability to complete functional and QoL testing needed to be evaluated. Our data show that the provision of SPN + EN versus EN alone significantly increased calorie/protein delivery over the first ICU week versus EN alone. Further, consistent encouraging trends in hospital mortality, ICU mortality, and QoL and functional endpoints (with no increased infection risk from PN) indicates a full-scale trial of SPN in high-nutritional-risk ICU patients focused on those with a NUTRIC score ≥5 regardless of BMI is indicated and has the potential to change practice by clarifying an objective measure of malnutrition to guide optimal use of SPN. It may also be optimal to focus a future trial in the more poorly EN-fed surgical ICU setting. Assuming we can carefully select sites and address patient ability to complete follow-up functional and QoL data, we propose that this future trial focus on a functional and/or QoL endpoint rather than mortality as its primary outcome.

## Additional file

Additional file 1: Table S1. Calorie and protein delivery by ICU type. **Table S2.** Calorie and protein delivery by BMI group. (DOCX 42 kb)

## Abbreviations

BMI: Body mass index
EN: Enteral nutrition
ICU: Intensive care unit
PICS: Post-intensive care syndrome
PN: Parenteral nutrition
QoL: Quality of life
SF-36: 36-Item Short Form Health Survey
SPN: Supplemental parenteral nutrition
