# Supplementary material for: A randomized trial of supplemental parenteral nutrition in underweight and overweight critically ill patients: the TOP-UP pilot trial
Source: Crit Care. 2017 Jun 9;21:142. doi: 10.1186/s13054-017-1736-8 (PMC5466764; doi:10.1186/s13054-017-1736-8)
Supplement: Additional file 1: Table S1. — Calorie and protein delivery by ICU type. Table S2. Calorie and protein delivery by BMI group. (DOCX 42 kb) [file 13054_2017_1736_MOESM1_ESM.docx]

**SUPPLEMENTAL DATA**

**Table S1:** **Calorie and Protein Delivery by ICU Type**

| **Table 1A: Surgical ICU** |  | |  |  |  |
| --- | --- | --- | --- | --- | --- |
|  | **EN Only (n=29)** | | **SPN+EN (OLIMEL)**  **(n=19)** | **Difference mean, %**  **(95% CI)** | **P-value** |
| Evaluable days | 10±7 | | 15±10 | 5 (-0 to 10) | 0.059 |
| Evaluable days in first week | 6±2 | | 6±2 | 1 (-0 to 2) | 0.282 |
| Calorie prescription | 1987±382 | | 1806±582 | -181 (-490 to 129) | 0.242 |
| Protein prescription | 118±26 | | 106±35 | -13 (-32 to 7) | 0.187 |
| EN only |  | |  |  |  |
| Calories first 27 days | 61±29 | | 58±23 | -3 (-19 to 12) | 0.668 |
| Calories first 7 days | 56±29 | | 58±26 | 2 (-14 to 19) | 0.79 |
| Protein first 27 days | 60±28 | | 57±25 | -3 (-19 to 13) | 0.697 |
| Protein first 7 days | 55±29 | | 58±29 | 2 (-15 to 20) | 0.784 |
| PN+EN |  | |  |  |  |
| Calories first 27 days | 64±27 | | 88±20 | 24 (10 to 38) | 0.001 |
| Calories first 7 days | 56±29 | | 95±18 | 38 (24 to 52) | <.001 |
| Protein first 27 days | 63±27 | | 84±22 | 21 (6 to 35) | 0.006 |
| Protein first 7 days | 56±29 | | 91±18 | 35 (21 to 49) | <.001 |
| **Table 1B: Medical ICU** |  |  | |  |  |
|  | **EN Only (n=42)** | **SPN+EN (OLIMEL)**  **(n=31)** | | **Difference mean, %**  **(95% CI)** | **P-value** |
| Evaluable days | 11±8 | 9±5 | | -3 (-6 to 1) | 0.103 |
| Evaluable days in first week | 6±2 | 6±2 | | -0 (-1 to 0) | 0.387 |
| Calorie prescription | 1746±421 | 1680±335 | | -66 (-243 to 111) | 0.459 |
| Protein prescription | 97±30 | 97±29 | | -0 (-14 to 13) | 0.959 |
| EN only |  |  | |  |  |
| Calories first 27 days | 76±23 | 73±24 | | -4 (-15 to 8) | 0.532 |
| Calories first 7 days | 77±24 | 73±25 | | -4 (-15 to 8) | 0.542 |
| Protein first 27 days | 70±24 | 62±21 | | -7 (-18 to 3) | 0.171 |
| Protein first 7 days | 69±22 | 63±22 | | -7 (-17 to 4) | 0.211 |
| PN+EN |  |  | |  |  |
| Calories first 27 days | 78±22 | 92±12 | | 14 (6 to 22) | 0.001 |
| Calories first 7 days | 78±24 | 95±9 | | 18 (10 to 26) | <.001 |
| Protein first 27 days | 71±23 | 80±17 | | 9 (-1 to 18) | 0.065 |
| Protein first 7 days | 70±22 | 83±15 | | 13 (4 to 21) | 0.003 |

Values are means ± standard deviations, unless noted otherwise. P-values and 95% CIs were calculated by the independent t-test for unequal variance.

Only days after the date of randomization and before the date of ICU discharge or death are considered evaluable days. Days where oral feeding was indicated as the reason for not receiving EN or PN have also been excluded. Two patients randomized to the EN arm and three patients randomized to the SPN+EN arm had no evaluable days and are thus excluded from this analysis. All calories exclude propofol but include protein supplementation. PN includes both study PN and non-study PN.

Abbreviations: CI, confidence interval; EN, enteral nutrition; ICU, intensive care unit; PN, parenteral nutrition; SPN, supplemental parenteral nutrition.

**Table S2:** **Calorie and Protein Delivery by BMI Group**

| **Table 2A: BMI <25** |  |  |  |  |
| --- | --- | --- | --- | --- |
|  | **EN Only (n=37)** | **SPN+EN (OLIMEL)**  **(n=26)** | **Difference mean, %**  **(95% CI)** | **P-value** |
| Evaluable days | 10±7 | 11±8 | 1 (-3 to 5) | 0.648 |
| Evaluable days in first week | 6±2 | 6±2 | -0 (-1 to 1) | 0.545 |
| Calorie prescription | 1672±335 | 1606±458 | -65 (-278 to 147) | 0.538 |
| Protein prescription | 91±23 | 87±29 | -4 (-18 to 10) | 0.562 |
| EN only |  |  |  |  |
| Calories first 27 days | 68±32 | 70±25 | 2 (-12 to 17) | 0.745 |
| Calories first 7 days | 68±31 | 66±30 | -2 (-18 to 14) | 0.816 |
| Protein first 27 days | 65±32 | 64±25 | -1 (-16 to 13) | 0.862 |
| Protein first 7 days | 65±30 | 61±29 | -4 (-19 to 12) | 0.638 |
| PN+EN |  |  |  |  |
| Calories first 27 days | 72±29 | 86±18 | 15 (3 to 27) | 0.018 |
| Calories first 7 days | 70±31 | 91±15 | 21 (9 to 33) | <.001 |
| Protein first 27 days | 69±30 | 79±21 | 10 (-3 to 23) | 0.132 |
| Protein first 7 days | 66±30 | 84±19 | 18 (6 to 31) | 0.005 |

| **Table 2B: BMI >35** |  |  |  |  |
| --- | --- | --- | --- | --- |
|  | **EN Only (n=34)** | **SPN+EN (OLIMEL)**  **(n=24)** | **Difference mean, %**  **(95% CI)** | **P-value** |
| Evaluable days | 11±8 | 11±8 | -0 (-4 to 4) | 0.958 |
| Evaluable days in first week | 6±2 | 6±1 | 0 (-1 to 1) | 0.468 |
| Calorie prescription | 2032±426 | 1860±397 | -173 (-391 to 46) | 0.120 |
| Protein prescription | 122±28 | 114±27 | -8 (-22 to 7) | 0.311 |
| EN only |  |  |  |  |
| Calories first 27 days | 72±20 | 64±24 | -8 (-20 to 4) | 0.171 |
| Calories first 7 days | 68±24 | 69±24 | 1 (-12 to 13) | 0.915 |
| Protein first 27 days | 66±17 | 56±20 | -10 (-20 to 1) | 0.062 |
| Protein first 7 days | 62±21 | 61±19 | -2 (-12 to 9) | 0.754 |
| PN+EN |  |  |  |  |
| Calories first 27 days | 73±20 | 94±11 | 22 (13 to 30) | <.001 |
| Calories first 7 days | 68±24 | 99±10 | 31 (22 to 40) | <.001 |
| Protein first 27 days | 66±18 | 84±16 | 17 (8 to 26) | <.001 |
| Protein first 7 days | 62±21 | 87±13 | 25 (16 to 34) | <.001 |

Values are means ± standard deviations, unless noted otherwise. P-values and 95% CIs were calculated by the independent t-test for unequal variance.

Only days after the date of randomization and before the date of ICU discharge or death are considered evaluable days. Days where oral feeding was indicated as the reason for not receiving EN or PN have also been excluded. Two patients randomized to the EN arm and three patients randomized to the SPN+EN arm had no evaluable days and are thus excluded from this analysis. All calories exclude propofol but include protein supplementation. PN includes both study PN and non-study PN.

Abbreviations: BMI, body mass index; CI, confidence interval; EN, enteral nutrition; PN, parenteral nutrition; SPN, supplemental parenteral nutrition.
